# An extremely rare case of feeding jejunostomy proceeding to intussusception

**DOI:** 10.1002/ccr3.7460

**Published:** 2023-06-14

**Authors:** Taraka Krishna Nulukurthi, L. V. Simhachalam Kutikuppala, Sai Kiran Kuchana, Mihnea‐Alexandru Găman, Samrat Babu Koirala, Sri Harsha Boppana

**Affiliations:** ^1^ Department of General Surgery Konaseema Institute of Medical Sciences and Research Foundation (KIMS&RF) Amalapuram India; ^2^ Department of General Surgery Dr YSR University of Health Sciences Vijayawada India; ^3^ Department of General Surgery Kakatiya Medical College Warangal India; ^4^ Faculty of Medicine Carol Davila University of Medicine and Pharmacy Bucharest Romania; ^5^ Department of Hematology, Center of Hematology and Bone Marrow Transplantation Fundeni Clinical Institute Bucharest Romania; ^6^ Nepalese Army Institute of Health sciences College of Medicine Kathmandu Nepal; ^7^ Division of Perioperative Informatics/Department of Biomedical, Informatics/Division of Regional Anesthesia, Department of Anaesthesiology University of California, San Diego La Jolla California USA

**Keywords:** carcinoma (CA) oesophagus, feeding jejunostomy, intussusception, jejunum

## Abstract

**Key Clinical Message:**

Feeding jejunostomy (FJ) is one of the frequently performed surgical procedures for enteral nutrition, but intussusception a very rare complication with quite challenging clinical outcome. It symbolizes a surgical emergency requiring prompt diagnosis.

**Abstract:**

Feeding jejunostomy (FJ) is a minor surgical intervention, which might lead to consequences that are potentially fatal. Mechanical issues such as infection, tube dislocation or migration, electrolyte and fluid imbalances, as well as complaints of gastrointestinal tract, are the most frequent consequences. A 76‐year‐old female, who is a known case of carcinoma (CA) esophagus: Stage 4 with Eastern Cooperative Oncology Group (ECOG) Class 3 presented with complaints of difficulty in swallowing and vomiting. As a part of palliative treatment, FJ is done and patient was discharged on postoperative day (POD) 2. Patient again presented to emergency department after 2 months with complaints of pain abdomen, unable to pass flatus and stools for 2 days. Contrast‐enhanced computed tomography was done, which revealed intussusception of jejunum with lead point as tip of feeding tube. Intussusception of jejunal loops is noted 20 centimeters distal to the site of insertion of FJ tube with tip of feeding tube as lead point. Reduction of bowel loops was achieved by gentle compression of distal part and are found to be viable. FJ tube was then removed and repositioned after which the obstruction got relieved. Intussusception is an extremely rare complication of FJ, where the clinical presentation can be likely to the various causes of small bowel obstruction. The fatal complications like intussusception in FJ can be prevented by remembering some technical considerations, such as attaching a 4–5 cm segment of the jejunum to the abdominal wall rather than a single‐point fixation and maintaining a minimum distance of 15 cm between the duodenojejunal (DJ) flexure and the FJ site.

## 1 INTRODUCTION

Feeding jejunostomy (FJ) is a minor surgical intervention, which might lead to consequences that are potentially fatal. Mechanical issues such as infection, tube dislocation or migration, electrolyte and fluid imbalances, as well as complaints of gastrointestinal tract, are the most frequent consequences.^1^ Adults who have intestinal intussusception on a jejunostomy tube experience this extremely uncommon complication very infrequently. It symbolizes a surgical emergency that calls for prompt diagnosis and treatment, which is frequently surgical, as we demonstrate in this instance.^2^ In order to provide nutrition to individuals who are unable to take it orally owing to illnesses affecting the stomach, esophagus, pancreas, duodenum, liver, or biliary system, a tube is put in the lumen of the proximal jejunum.^3^ Mechanical, bacterial, gastrointestinal, or metabolic problems have been reported with jejunostomies. “Jejunostomy tube‐induced intussusception” (JTI), an extremely uncommon complication with a less than 1% incidence, is one of many.^4^ It could be momentary, intermittent, or irreducible. Typically, the patient will appear with nausea, a lump in the belly, or both.^5^ We discuss a case of an extremely rare case of FJ proceeding to intussusception which is very scarcely reported before, along with the surgical treatment that followed.

## 2 CASE REPORT

A 76‐year‐old female, who is a known case of carcinoma (CA) esophagus: Stage 4 with Eastern Cooperative Oncology Group (ECOG) Class 3 presented with complaints of difficulty in swallowing, vomiting's for both solids and liquids. As a part of palliative treatment, FJ is done and patient was discharged on postoperative day (POD) 2. Patient again presented to emergency department after 2 months with complaints of pain abdomen, unable to pass flatus and stools for 2 days.

**FIGURE 1:**
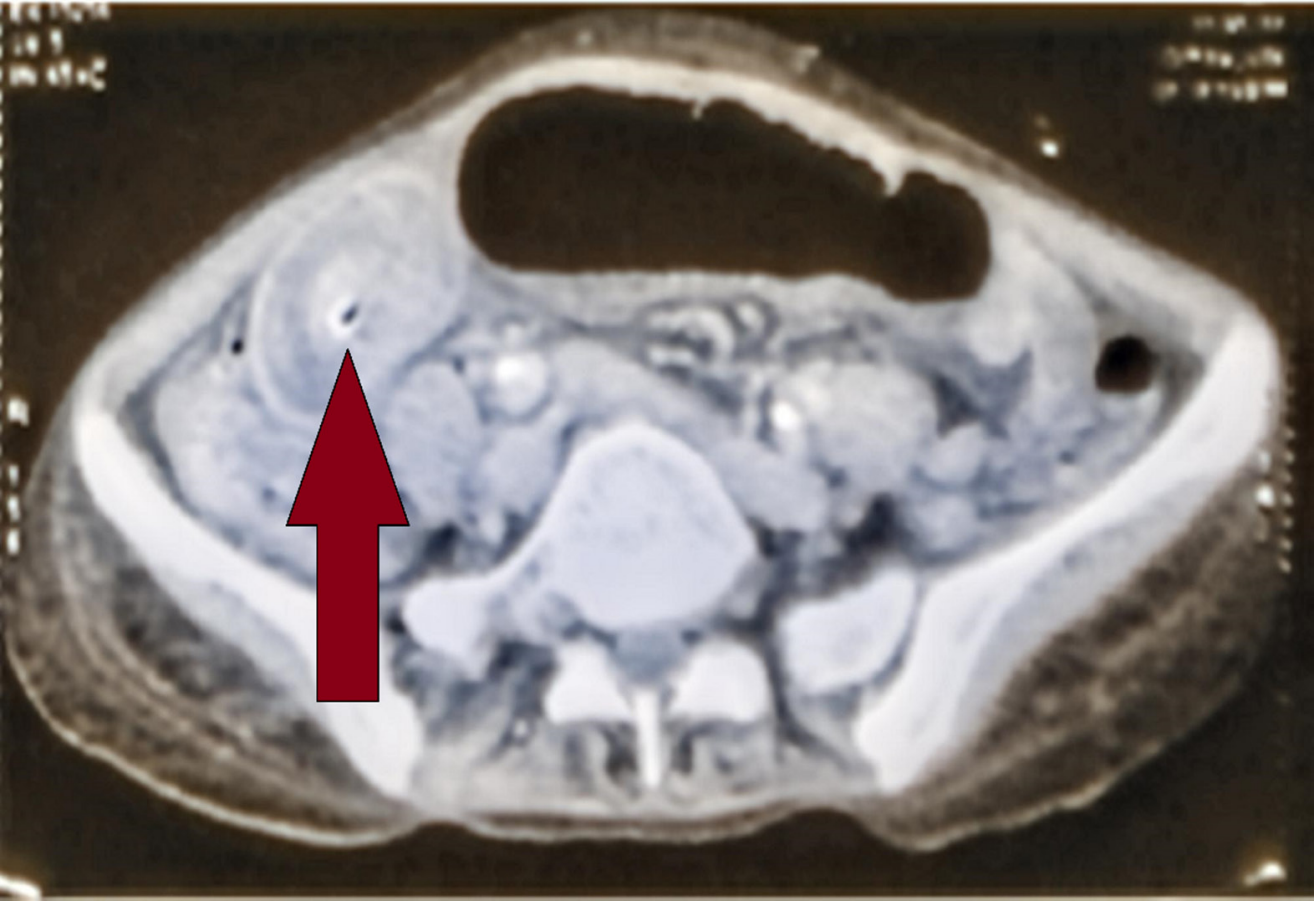
CECT image showing target sign suggestive of intussusception.

**FIGURE 2:**
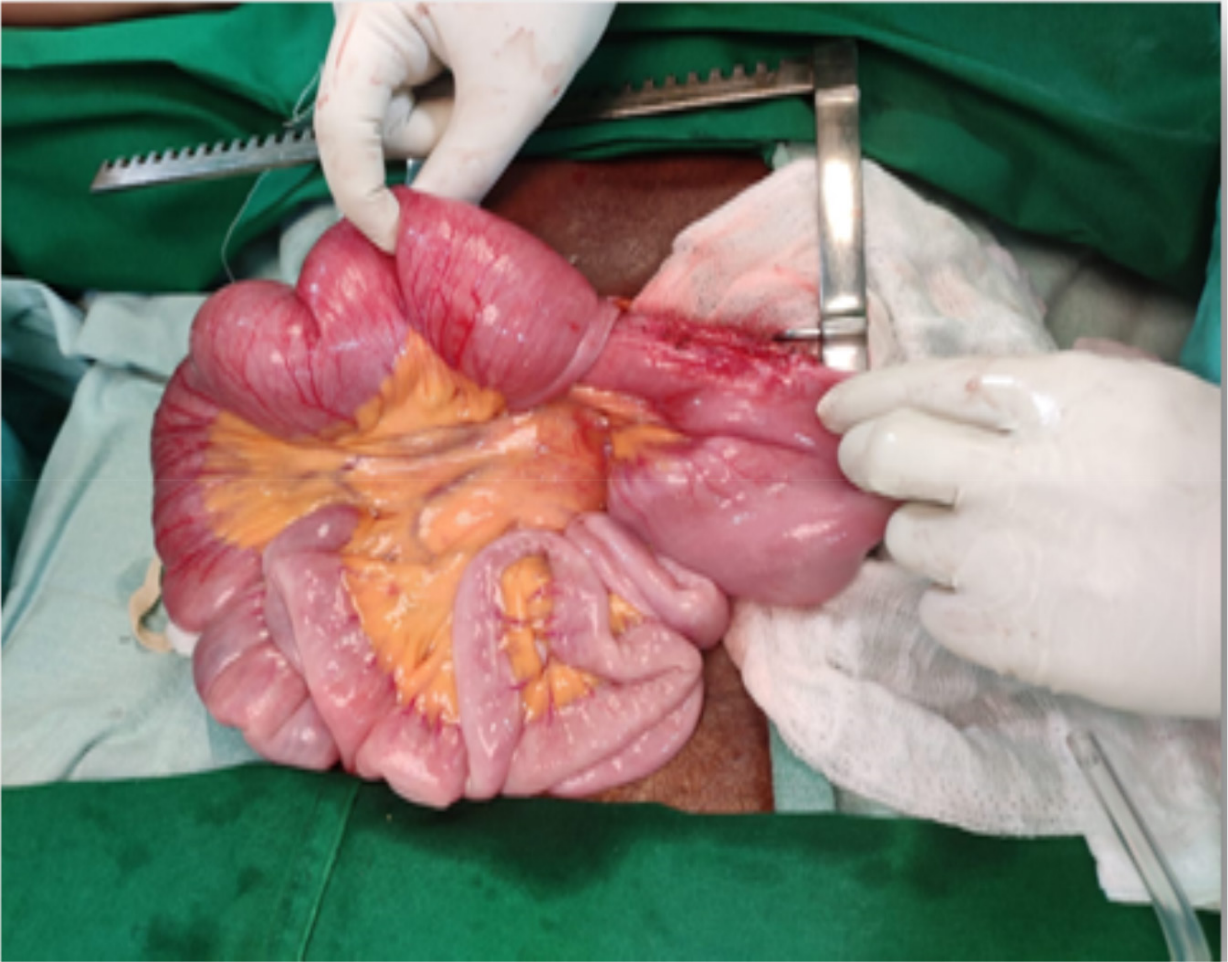
Image showing intussusception of jejunum with lead point as tip of feeding tube.

On clinical examination, abdominal distension present, diffused abdominal tenderness present with guarding and rigidity. Regurgitation of contents through the feeding tube noted during feeding. Tympanic note on percussion all over the abdomen. Contrast Enhanced Computed Tomography (CECT) was done, which revealed intussusception of jejunum with lead point as tip of feeding tube (Figure 1). Patient was shifted to Operation Theatre and Emergency Laparotomy was done. Intussusception of jejunal loops is noted 20 cm distal to the site of insertion of FJ tube with tip of feeding tube as lead point (Figure 2). Reduction of bowel loops was achieved by gentle compression of distal part and are found to be viable. FJ tube was removed and repositioned. Obstruction was relieved. Patient was kept in intensive care unit (ICU) for 2 days, feeding through FJ tube were initiated on POD 1, patient was stable and was discharged on POD 5. The provisional diagnosis was acute intestinal obstruction. The differential diagnosis for the case were malignancy, stricture, and adhesions.

## 3 DISCUSSION

Feeding jejunostomy is one of the frequently performed surgical procedures for enteral nutrition. The clinical presentation of intussusception, a very rare complication of FJ, may resemble that of other conditions that result in small bowel blockage.^6^, ^7^ Since it may not interfere with tube feeding, the diagnosis of intussusception caused by a jejunostomy tube is challenging. Clinical, radiographic, and endoscopic findings may not differ from those of adhesions or other small intestinal obstruction causes. The diagnosis is typically confirmed by ultrasonogram (USG) and computed tomography (CT) scan.^8^ Jejunostomy problems might be physical, infectious, gastrointestinal, or metabolic. Kinking, coiling, malpositioning, knotting, blockage of the tube, and retrograde flow are examples of mechanical issues with tubes. Infectious side effects include peritubal biliary leakage and wound infection.^9^ Small bowel obstruction, extraluminal tracks or collections, nonobstructive small bowel constriction, intussusceptions, and jejunal hematomas are some of the gastrointestinal problems. Hypokalemia, hyperglycemia, hypomagnesemia, hypophosphatemia, electrolyte, and water imbalance are the metabolic side effects.^10^ Jejunostomy tube‐induced intussusception is a very uncommon complication of FJ with an incidence of 0.01%.^11^ Bilious vomiting, pain, and occasionally a transitory lump in the abdomen are common presentations. It is most frequently observed in men, young new‐borns, and when the distal pigtail on the tube is present.^12^ Intussusception caused by a jejunostomy tube typically goes away on its own, but occasionally surgery may be necessary. It is possible to try reduction by injecting air or contrast through the tube.^6^ Operative intervention is necessary if a conservative approach fails to resolve the issue. If the bowel is not ischemic, surgical reduction can be used to treat it. If there is stenosis, perforation, or gangrene, resection is suggested. Following surgery, the feeding tube may be utilized without risking a recurrence of intussusception.^9^

## 4 CONCLUSION

It may be possible to prevent this complication by remembering some technical considerations, such as attaching a 4–5 cm segment of the jejunum to the abdominal wall rather than a single point fixation and maintaining a minimum distance of 15 cm between the duodenojejunal flexure and the FJ site.

## AUTHOR CONTRIBUTIONS

**Taraka Krishna Nulukurthi:** Conceptualization; data curation; investigation; writing – original draft. **L V Simhachalam Kutikuppala:** Investigation; methodology; validation; writing – original draft; writing – review and editing. **Sai Kiran Kuchana:** Writing – original draft; writing – review and editing. **Mihnea‐Alexandru Găman:** Validation; writing – original draft; writing – review and editing. **Samrat Babu Koirala:** Resources; writing – original draft; writing – review and editing. **Sri Harsha Boppana:** Writing – original draft; writing – review and editing.

## CONFLICT OF INTEREST STATEMENT

The authors have no conflict of interest to declare.

## CONSENT

Written informed consent was obtained from the patient to publish this report in accordance with the journals patient consent policy.

## Data Availability

Not applicable.
